# Mindsets and Self-Concepts About Self-Regulated Learning: Their Relationships With Emotions, Strategy Knowledge, and Academic Achievement

**DOI:** 10.3389/fpsyg.2021.661142

**Published:** 2021-06-18

**Authors:** Yves Karlen, Carmen Nadja Hirt, Alina Liska, Ferdinand Stebner

**Affiliations:** ^1^School of Education, University of Applied Sciences and Arts Northwestern Switzerland, Windisch, Switzerland; ^2^Institute of Educational Research, Ruhr University Bochum, Bochum, Germany; ^3^Institute of Educational Science, University of Osnabrück, Osnabrück, Germany

**Keywords:** implicit theories, mindsets, self-concept, self-regulated learning, strategy knowledge, metacognition, emotion

## Abstract

Being a self-regulated learner and believing that deliberate strategy use might be an effective way of overcoming learning challenges is important for achieving academic success. Learners' self-theories about their abilities might explain why some students are more inclined to engage in self-regulated learning (SRL) than others. This study aims to investigate the relationships between students' mindsets and self-concepts about SRL and their correlation with enjoyment, boredom, strategy knowledge, and academic achievements. As covariates, we included gender, age, and academic track. We surveyed 244 students (46.3% female) from the lower secondary school level with a mean age of 14.57 years. The results revealed that mindsets about SRL support more adaptive learning emotions (i.e., higher enjoyment and lower boredom) and positively relate to students' strategy knowledge. The students' self-concepts about SRL are positively related to their enjoyment and academic achievements. Gender-specific differences between the students revealed a disadvantage for the boys, who had lower self-concepts about SRL, lower strategy knowledge, and lower academic achievements in comparison to the girls. Furthermore, the study also revealed that students in the lower academic track adhered more to a fixed mindset about SRL and had lower strategy knowledge than their peers in the higher academic track. Finally, we found an indirect relationship between mindset about SRL and academic achievement via self-concepts about SRL. Overall, our results emphasize the importance of students' mindsets and self-concepts about SRL for their learning and academic achievements.

## Introduction

Self-regulated learners are agents of their learning. They know when and how to use strategies effectively to overcome challenges, they are reflective, motivated, and strategic, and they believe that abilities in self-regulated learning (SRL) will help them succeed in school and beyond (Pressley et al., 1987). However, some students do not believe that strategies are necessary for learning or that they might be an effective way of overcoming learning challenges. Instead, they believe that if a person has high abilities, one does not need deliberate strategies to master obstacles (Hertel and Karlen, 2020). Students might also believe that they do not have sufficient abilities in SRL and therefore do not apply strategies. Learners' self-theories about their abilities play an essential role in the context of academic learning and can explain different patterns of emotions, motivation, strategy use, persistence in SRL, response to challenges and setbacks, and academic achievement (Dweck and Leggett, 1988; Efklides, 2011; Karlen et al., 2019; Lawson et al., 2019). Vosniadou et al. (2020) argued that it might be beneficial to examine beliefs not as isolated units but as connected to other beliefs and to other cognitive and emotional structures. Therefore, forming a belief system, which is critical for learners perception, and interpretation of the learning context and prediction of their learning behavior (for an overview of beliefs about SRL see Lawson et al., 2019). Two core self-theories about abilities are learners' implicit theories about the nature of abilities (mindsets) as trait-like (fixed mindset) or malleable (growth mindset) and their self-theories about the level of one's abilities, also called self-concept (Dweck and Leggett, 1988; Bong and Skaalvik, 2003). These beliefs represent independent but related components of individuals' self-related implicit beliefs that are related to various motivational and cognitive effects such as better emotional, motivational, and metacognitive self-regulation or adaptive management of challenges (Ommundsen et al., 2005; Pekrun, 2006; King et al., 2012; Van der Beek et al., 2017; Yeager and Dweck, 2020).

Individuals can hold different mindsets and self-concepts about different abilities (Gunderson et al., 2017). The literature that focuses on mindsets and self-concepts about SRL is still scarce and requires further examination (Chen et al., 2020; Hertel and Karlen, 2020). Our study builds upon existing empirical work that links motivational and affective aspects of learning to SRL and students' academic achievement (Burnette et al., 2013). Focusing on mindsets and self-concepts about SRL may offer a key to understanding how much learners are inclined to engage in SRL and, in turn, develop their SRL competencies (Efklides, 2011; Vosniadou et al., 2020). So far, little is known about self-theories about SRL and their possible relationship to enjoyment or boredom in learning. This study focuses on boredom and enjoyment, as researchers showed that these emotions are of particular relevance for students' learning and academic achievement (Camacho-Morles et al., 2021). Hence, this paper aims to examine the role of students' mindsets and self-concepts about SRL concerning their enjoyment and boredom about learning, strategy knowledge, and academic achievement.

## Mindsets and Self-concepts about Self-regulated Learning

Individuals can hold different self-theories about their abilities, which create a system of meaning that affects how individuals approach academic situations, how they perceive their knowledge and abilities, how they self-regulate their learning, and how they interpret and respond to challenges within such situations (Bong and Skaalvik, 2003; Efklides, 2011; Lawson et al., 2019; Yeager and Dweck, 2020). By following Carol Dweck's social cognitive theory (Dweck and Leggett, 1988), we focus on students' implicit theories (mindsets) about the nature of their abilities as trait-like (fixed mindsets) or malleable (growth mindsets). Students who adhere to a fixed mindset believe that their abilities are relatively pre-determined, like a fixed talent that is incapable of developing. Accordingly, they are more likely to demonstrate maladaptive learning behaviors such as withdrawing when challenges arise, engaging in procrastination, and avoiding expending effort and negative evaluations of their abilities since these could indicate that they possess low levels of talent (Burnette et al., 2013; Haimovitz and Dweck, 2017). In contrast, students who adhere to a growth mindset tend to perceive learning situations as opportunities to grow and expand their competencies. Thus, mindsets are associated with individual differences in academic achievements. However, two meta-analyses recently revealed small effect sizes between implicit theories of intelligence and academic achievement (Costa and Faria, 2018; Sisk et al., 2018). Mindsets may indirectly affect academic achievement since they foster more adaptive learning behaviors (Burnette et al., 2013; Karlen et al., 2019). By transferring implicit theories of intelligence to an SRL context, students who adhere to a growth mindset about SRL assume that competencies in SRL can be learned and improved through practice, effort, and training. In contrast, students who adhere to a fixed theory about SRL suppose that SRL competencies are relatively stable over time and are related to a given talent (Hertel and Karlen, 2020). Mindsets about SRL are relevant since learning and engaging in SRL can be a strenuous process that requires perseverance and an adaptive way of dealing with challenges. Moreover, having a strategic repertoire is not a guarantee that one will select and use those strategies wisely. Thus, a highly developed repertoire might not always support learning as it is expected to Carr and Taasoobshirazi (2017) and Parkinson and Dinsmore (2018). It is more important to believe that with practice and experience, strategies will become more effective. SRL requires not only a broad strategy repertoire and knowledge about those strategies but also self-confidence and the belief that with practice, time, and effort, SRL will increase academic achievement (Efklides, 2011). In this respect, mindsets are significant for students who wish to gain competencies in SRL and are also addressed in SRL training sessions and interventions (Chen et al., 2020; Hertel and Karlen, 2020).

Self-concept beliefs are relatively stable, multidimensional, and hierarchical cognitive representations of one's perceived level of academic abilities in general and in different academic domains (Bong and Skaalvik, 2003). These mental representations of individuals' abilities include self-descriptions and self-evaluations (Brunner et al., 2010). One's self-concept is formed through past experiences and comparisons and is continually reinforced by evaluative inferences (Möller et al., 2020). Self-concepts about various domains positively associate with persistence, positive emotions about learning, effort, strategy use, and academic achievements in those domains, as well as long-term educational attainments (Gogol et al., 2017; Möller et al., 2020). However, people differ from each other in terms of their self-concepts. They can have different self-concept levels in different domains (Brunner et al., 2010). Learners also might differ from each other in terms of their self-concepts about SRL. In contrast to individuals with low self-concepts about SRL, individuals with high self-concepts about SRL are convinced that they are good at SRL and can achieve their desired learning goals through strategic learning.

Mindsets and self-concepts typically demonstrate weak correlations (Ommundsen et al., 2005; Cury et al., 2006; Kornilova et al., 2009). They are largely independent of each other since individuals who adhere to fixed or growth mindset can have high or low self-concepts in a specific domain. We know from various studies that mindsets and self-concepts make independent contributions that explain adaptive and maladaptive behaviors, even after controlling for each other (Ommundsen et al., 2005). For self-concepts, slightly stronger relationships have been found between students learning behaviors and various emotional, motivational, and cognitive outcomes than between implicit theories and these same factors (Ommundsen et al., 2005; Kornilova et al., 2009; Priess-Groben and Hyde, 2017). However, Dweck and Leggett (1988) suggested in their theoretical model that mindsets take on a protective function for students' self-concepts. Students who adhere to a growth mindset might see mistakes as feedback regarding skills that are not yet sufficiently available but can be developed. Thus, failures will likely not damage their self-concepts as much as they would for students who adhere to a fixed mindset. For example, Robins and Pals (2002) reported that students who adhere to a fixed mindset experience a decline in self-esteem (a self-concept-related construct) during college, whereas students who adhere to a growth mindset increase their self-esteem.

## Mindsets, Self-concepts, and Emotions in Self-regulated Learning

Achievement emotions are linked to achievement-related activities and outcomes and comprise subjective feelings and psychological, cognitive, expressive, and motivational components (Pekrun, 2006). Based on Pekrun's (2006) control-value theory and Eflikdes' (2011) “metacognitive and affective model of self-regulated learning,” mindsets and self-concepts function as motivational resources that are essential antecedents for emotions in academic learning and guide SRL processes (Gogol et al., 2017; Van der Beek et al., 2017; Bakadorova et al., 2020). Mindsets and self-concepts refer to a control-related appraisal component, which influences the regulation of emotions (Pekrun, 2006; Efklides, 2011). Accordingly, the perception that one controls the learning process and its outcomes should promote enjoyment and reduces boredom while learning (Pekrun and Stephens, 2010). Students who adhere to growth mindsets see success as controllable and, thus, would rather perceive enjoyment. In contrast, the lack of control that students who adhere to a fixed mindset might experience in challenging situations is associated with the experience of anxiety or boredom while learning (King et al., 2012; Lou and Noels, 2020). In their meta-analysis, Burnette et al. (2013) found a negative link between a growth mindset about intelligence and negative emotions, which means that students with a growth mindset experience fewer negative emotions about learning. From a theoretical and empirical perspective, one can expect to see a similar correlational pattern between self-concepts and achievement emotions. Students should enjoy learning when they judge themselves as being competent enough to master a learning task. In contrast, boredom should result when perceived competence and control are low. Empirical findings support those theoretical assumptions and showed that students who feel competent in a domain perceive a higher level of control over learning and achievement activities, which leads to higher enjoyment and less boredom (Pekrun and Stephens, 2010; Van der Beek et al., 2017).

## Mindsets, Self-concepts, Emotions, and Their Relationship to Strategy Knowledge

Successful self-regulated learners are characterized by broad strategy repertoires, a high level of strategy knowledge, joy of learning, and motivation that supports in-depth and persistent SRL (Pressley et al., 1987; Pekrun et al., 2002). Besides motivation and beliefs that support the use of strategies, learners particularly need metacognitive knowledge to process achievement tasks and situations in a goal-oriented manner (Karlen et al., 2014; Ben-Eliyahu and Linnenbrink-Garcia, 2015; Lawson et al., 2019). Metacognition has been broadly defined as knowledge about cognition and the regulation and monitoring of cognitive functions (Flavell et al., 2002; Pintrich, 2002). On the one hand, this conceptualization includes executive metacognitive skills that are related to planning, monitoring, and regulating one's activities. On the other hand, it refers to learners' knowledge about their information-processing skills, the nature of tasks, and strategies for coping with such tasks (Paris et al., 1983; Pressley et al., 1987). Strategy knowledge comprises declarative knowledge (i.e., knowing about the existence of strategies), procedural knowledge (i.e., knowing about how a strategy can be effectively used), and conditional knowledge (i.e., knowing when and why strategies are useful for completing a specific task) (Paris et al., 1983). Thus, strategy knowledge includes knowledge about the effectiveness of a strategy, the range of its appropriate applications, and how to use it to accomplish various tasks (Pressley et al., 1987). Researchers have linked strategy knowledge to the effective use of strategies and higher achievement in various domains (Händel et al., 2013; Maag Merki et al., 2013).

Efklides (2011) has included mindsets, self-concepts, and emotions at the personal level that set goal-directed top-down and bottom-up SRL processes and are closely linked to student's metacognition. The relationship between mindsets and SRL has predominantly been investigated using mindsets about intelligence. In comparison to those who adhere to a growth mindset about intelligence, students who adhere to a fixed theory of intelligence are more likely to fail to employ metacognitive skills, which leads to higher levels of procrastination, worse time management, the decreased use of strategies, negative emotional regulation, and failure (Burnette et al., 2013; Yan et al., 2014). Initial research groups recently linked mindsets to SRL. Chen et al. (2020) found that mindsets about SRL, which they called “strategic mindsets,” positively relate to the use of metacognitive strategies and academic achievement. Hertel and Karlen (2020) compared the predictive powers of mindsets about intelligence and mindsets about SRL regarding SRL. They found that mindsets about SRL more strongly relate to students' learning goals, self-reported strategy use, and strategy knowledge than mindsets about intelligence do.

So far, the specific link between students' self-concepts about SRL and their SRL has not been examined. Nevertheless, when focusing on students' academic self-concepts, researchers have found empirical evidence that supports a positive relationship between academic self-concepts and SRL. Bakadorova et al. (2020) found that high school students' academic self-concepts positively associate with emotional engagement (enjoyment of learning) and behavioral school engagement (i.e., involving a student's persistence in accomplishing tasks, attention during a lesson, or effort expended). In a study that was conducted with first graders, Roebers et al. (2012) reported that the students' domain-specific self-concepts were substantially associated with metacognitive monitoring. Finally, in a study that was conducted with kindergarteners, Compagnoni and Losenno (2020) found that their academic self-concepts positively related to their behavioral self-regulation.

Researchers have suggested that emotions can have a profound and long-term influence on students' metacognition because they favor engagement in SRL and the use of different strategies (Perry et al., 2001; Pekrun and Stephens, 2010). Several studies have demonstrated that while positive emotions such as enjoyment promote the use of in-depth strategies and students' engagement in metacognitive processes (i.e., the activation of strategy knowledge and self-evaluation), negative, deactivating emotions such as boredom promote maladaptive SRL (Pekrun et al., 2002; Ben-Eliyahu and Linnenbrink-Garcia, 2015; Chatzistamatiou et al., 2015). Regulating negative emotions (e.g., boredom) and supporting positive emotions (e.g., enjoyment) should thus facilitate successful SRL and support long-term engagement in SRL (Pekrun et al., 2002; Ben-Eliyahu and Linnenbrink-Garcia, 2015). Empirical research also indicates that emotions are a significant aspect of successful learning processes that lead to higher academic achievement (Camacho-Morles et al., 2021). For example, Perry et al. (2001) showed in their longitudinal study that students with higher academic control reported less course boredom, were more motivated, used more strategies, and obtained higher course grades. Researchers assume that SRL mediates the effects of emotions on academic achievement (Ben-Eliyahu and Linnenbrink-Garcia, 2015).

## Gender, Age, and Academic Track as Covariates of Self-regulated Learning

When focusing on students' genders, researchers have observed null or mixed gender differences in domain-general mindsets about intelligence (Compagnoni et al., 2019; Warren et al., 2019). Hertel and Karlen (2020) found no correlation between gender and mindsets about the malleability of SRL in a sample of university students. However, they discovered that girls more strongly believe that SRL is relevant for academic success in universities (i.e., mindsets about the relevance of SRL). Concerning self-concepts, gender-specific differences depend mainly on a subject's social attributions (i.e., math self-concepts are higher for male students) and might vary from domain to domain (Lauermann et al., 2019). However, researchers have repeatedly demonstrated that girls have higher strategy knowledge than boys and are thus more successful in SRL (Händel et al., 2013; Maag Merki et al., 2013), which might also positively influence their self-concepts about SRL over long periods. Concerning students' emotions, existing evidence has demonstrated that boys report less enjoyment and more boredom about learning than girls do (Pekrun et al., 2017; King and dela Rosa, 2019). As they age and experience more extended schooling, older students are more likely to report less enjoyment about learning and higher boredom than younger students are (Perry et al., 2001). Students in certain countries (e.g., Germany or Switzerland) are assigned to different types of schools with different academic requirements at the lower secondary level. Thus, students finish compulsory school in different academic tracks. One can expect students in a track with lower academic requirements to ascribe more of a fixed mindset and to have lower strategy knowledge than students in a track within higher academic requirements (Händel et al., 2013; Warren et al., 2019).

## Aims and Hypotheses of the Present Study

Theoretical concepts highlight how students' mindsets and self-concepts affect their emotions, engagement, and development in learning in general and in specific in SRL (Dweck and Leggett, 1988; Efklides, 2011). However, the literature that focuses on mindsets and self-concepts about SRL is still scarce. We aim to provide new insight into how more domain- or content-specific mindsets and self-concepts about SRL are related to each other (Research question 1). We specifically aim to examine the relationship between students' mindsets and self-concepts about SRL with their enjoyment, boredom, and strategy knowledge (Research question 2). Finally, we investigate how mindsets and self-concepts about SRL influence students' academic achievements when taking students' emotions and strategy knowledge into account (Research question 3). Based on the literature review presented in the previous sections, we hypothesized that mindsets and self-concepts about SRL would demonstrate a small but positive correlation (Hypothesis 1). We expected to find that mindsets and self-concepts about SRL would positively relate to enjoyment (Hypotheses 2a), negatively relate to boredom (Hypotheses 2b), and positively relate to strategy knowledge (Hypotheses 2c). Furthermore, we expected enjoyment to positively relate to strategy knowledge (Hypotheses 2d) and boredom to negatively relate to strategy knowledge (Hypotheses 2e). Finally, we expected students' mindsets and self-concepts about SRL to enhance their academic achievements because these facilitate students' strategy knowledge (Hypothesis 4).

## Methods

### Participants

The participants were lower secondary school students (*N* = 244; 46.3% female) from 13 different classes from one school district situated in a rural area in the German-speaking part of Switzerland. School principals decided that every class should participate in this survey. Therefore, participation was mandatory for all the classes. However, the parents had to consent before the study was conducted, and students were allowed to withdraw from the online survey at any time. Out of 281 students forty-one decided not to participate in this study.

In Switzerland, lower secondary schools are usually divided into two or three different school types with different academic tracks (performance-based levels). In our school district, students are grouped into two different academic tracks based on their elementary school report cards. The highest track is for the most gifted children and prepares students for university entrance. The low-medium track includes two groups of students, preparing them either for vocational education and training or continuing education in upper secondary schools. Most of the students who participated (71.3%) attended the low-medium academic track (students mixed into one class), and 28.7% attended the highest academic track, which roughly corresponded to the distribution of students to academic tracks (school type) in Switzerland. Lower secondary school lasts 3 years (seventh [*n* = 88], eighth [*n* = 83], and ninth [*n* = 73] grade). Students were, on average, *M* = 14.57 years old (*SD* = 0.94). Most students (87.6%) reported that both parents or one parent were born in Switzerland, while a few (12.4%) reported that neither of their parents were born in Switzerland. A majority of the students (85.1%) reported speaking the instructional language at home (Swiss German or German), followed by Portuguese with 2.9%, English and other languages with each 2.5%, Albanian with 2.0%, and Serbian and Turkish with each 1.2%. Only a small number of students reported speaking French, Italian, or Spanish, with each 0.8%. A minimal amount of the students did not indicate any language (0.8%).

### Measures

All students who participated in the study completed an online survey during class time. The average time to complete the questionnaire (*M* = 26.37 min., *SD* = 6.68; without instruction time was well within the allowed timeframe of one lesson (45 min). The questionnaire was written in German, as it is the official instructional language. Throughout the questionnaire, we used the term “self-organized learning” instead of the term “self-regulated learning” as students were more familiar with the first term. In Switzerland, the term self-organized learning has become more established in schools. It is a pedagogical term that includes our understanding of the scientific term SRL. Nevertheless, both terms are used synonymously. The descriptive statistics and internal reliabilities for each variable are presented in Table 1.

**Table 1:** Descriptive statistics, internal reliabilities, and correlations.

**Variables**	**Cronbach's α**	***n***	***M***	***SD***	**Range**	**1**	**2**	**3**	**4**	**5**	**6**	**7**	**8**	**9**
1. Mindsets about SRL	0.68	244	3.75	0.74	1.67–5.00	-	0.19^**^	0.26^***^	−0.19^**^	0.21^**^	0.14^*^	−0.07	−0.08	−0.15^*^
2. Self-concepts about SRL	0.87	244	4.30	1.05	1.00–6.00		-	0.26^**^	−0.21^***^	0.21^**^	0.32^***^	−0.21^***^	−0.12	−0.04
3. Enjoyment	0.90	243	3.37	1.09	1.00–6.00			-	−0.73^***^	0.31^***^	0.24^***^	−0.25^***^	−0.21^***^	−0.10
4. Boredom	0.79	244	3.24	1.03	1.25–6.00				-	−0.18^*^	−0.20^**^	0.26^***^	0.29^***^	0.15^*^
5. Strategy knowledge	-	225	3.47	2.07	0.00–10.00					-	0.31^***^	−0.43^***^	−0.16^*^	−0.28^***^
6. Academic achievement	-	244	4.58	0.44	3.55–5.60						-	−0.33^***^	−0.13	−0.13
7. Gender^a^	-	244	-	-	-							-	0.18^**^	0.15^*^
8. Age	-	244	14.57	0.94	12.83–17.00								-	0.27^***^
9. Academic track^b^	-	244	-	-	-									-

*M, mean, SD, standard deviation*.

a *Female = 1; male = 2*.

b *Higher academic track = 1; lower academic track = 2*.

* *p < 0.05*.

** *p < 0.01*.

*** *p < 0.001*.

#### Mindsets About Self-Regulated Learning

We used a validated scale from Hertel and Karlen (2020) to assess the students' mindset about SRL. The scale included three items that incorporated a five-fold scale [sample item: “Everyone has a certain ability to self-organize their learning, and this… (1) cannot be changed to (5) can be changed”]. Higher values represented stronger endorsements of a growth mindset, meaning that higher values indicated that the students more strongly believed in the malleability of SRL.

#### Self-Concepts About Self-Regulated Learning

The students' self-concepts about SRL were assessed using a three-item scale (Karlen et al., 2020). The scale consists of three items (sample item: “I am good at self-organizing my learning”). Each item was scored on a six-point scale from 1 (*does not apply at all*) to six (*entirely true*). A higher score indicated a higher self-concept about SRL.

#### Enjoyment and Boredom About Learning

Enjoyment and boredom about learning were measured using the Achievement Emotions Questionnaire (AEQ), which was developed by Pekrun et al. (2011). As the number of items in the questionnaire was limited, we used fewer items than what the original instruments included. Four items were used to assess enjoyment (sample item: “I enjoy acquiring new knowledge”), and four items were used to assess boredom (sample item: “I find learning to be rather boring”). Answers were provided on a six-point scale from 1 (*does not apply at all*) to 6 (*entirely true*).

#### Strategy Knowledge

The students' strategy knowledge was assessed using a newly developed vignette test that outlines a fictitious learning situation in which students are asked to describe their intended approaches to processing a given task. The vignette test is based on similar procedures that have successfully and validly captured strategy knowledge using a vignette or scenario-based procedure (e.g., Händel et al., 2013; Maag Merki et al., 2013). With such tests, not the frequency of strategy use across different learning situations is measured, but students' knowledge about the use of strategies for completing a specific learning task (Schuster et al., 2020). Such vignette tests make it possible to test learners' spontaneous recall of strategies in a relatively short time. These tests have higher validity than, for example, questionnaires measuring the retention of strategies because they are contextualized and instead measure the qualitative use of strategies than the frequency of strategy use in general (Wirth and Leutner, 2008).

The vignette test includes a typical school learning situation, which requires the use of different learning strategies: “Imagine a class is about to complete a major exam. Therefore, the teacher gives the class a great deal of content to learn for the next 2 weeks. What could the students do to make sure that their learning for the exam goes well? Please describe all your tricks and pieces of advice for learning successfully.” The students' descriptions, provided in an open-response format, were analyzed using a developed coding manual based on the categorization of strategies developed by Weinstein and Mayer (1986). Thus, the category system for coding the students' described strategies contained three main categories: cognitive strategies, metacognitive strategies, and resource management strategies. These main categories were further refined by the differentiation developed by Wild and Schiefele (1994). A distinction was made among cognitive learning strategies between rehearsal, organization, and elaboration strategies. For metacognitive strategies, we differentiated between planning, monitoring, reflection, and regulation strategies. The resource management strategies were divided into internal (emotions, motivation, effort, attention, time management) and external (help-seeking, learning environment, peer learning) resource management strategies.

The students' answers were coded according to the strategies that they recalled (quantitative approach) and the specific instructions that they provided for the practical use of these strategies (qualitative approach). All of the named strategies had to include a reference to the learning situation. For example, if a cognitive strategy that was related to the learning situation was mentioned (e.g., “I suggest using a text-marking strategy”), the students received one point. Moreover, if they also provided a suggestion that was related to the strategy's quality of use, a further point was awarded (e.g., “I suggest the text-marking strategy: the students should first read the paragraph, ask a question, and then highlight the answer”). Students received zero points if they did not mention any strategy. All points across all categories were added together to calculate the total score. There was no point limit. The maximum points to be achieved varied depending on the number of strategies each student named.

Two independent coders with expertise in SRL double-coded a subsample of the students' answers (*N* = 25). This subsample corresponded to approximately 10% of the total sample. Subsequently, Cohen's kappa was used to determine the observer agreement. Interrater reliability was good, with Cohen's κ = 0.87.

#### Academic Achievement

We assessed the students' levels of academic achievement using a mean score (i.e., their grade point averages) based on their subject-specific grades. We obtained the students' official grades from their report cards. As reflected by several report card grades, the averaged measure of the student's academic achievement is a reliable indicator of their overall school performance. In Switzerland, grades range from one to six; six indicate outstanding performance, and one indicates poor performance. Thus, higher numbers represented higher levels of academic achievement.

### Analysis

The data were analyzed using descriptive and correlational analyses that utilized SPSS Version 26 and Mplus 8.2 (Muthén and Muthén, 1998-2007). To make full use of the data, we applied the full information likelihood method (FIML). This procedure allowed us to include all available information to estimate the models. The average rate of missing values per variable was 1.13% (range: 0.0–7.8%). The maximum likelihood estimator (MLR) was used to ensure robustness to non-normality. First, to explore the dimensionality and the reliability of the scales of mindsets and self-concept about SRL, we performed a confirmatory factor analysis (CFA) with latent variables. Second, we conducted a path analysis to examine the relationship between all variables. To improve the number of free parameters in accordance with sample size ratios and increase the parameter estimates' stability, we used manifest variables instead of latent variables. Thanks to this approach, our path model met the minimal assumptions regarding the ratio of free parameters per case (Kline, 2016). We included gender, age, and academic track as covariates. The model fit indices were interpreted using several model fit indicators (Schermelleh-Engel et al., 2003): χ^2^/df ratio value (should be lower than 3), the root mean square error of approximation (RMSEA, should be lower than 0.06), the comparative fit index (CFI, should be higher than 0.95), and the standardized root mean square (SRMR, should be lower than 0.08). The indirect effects were examined using a bias-corrected bootstrapping procedure (MacKinnon et al., 2004). Since bootstrapping is not yet available for MLR estimation in Mplus, maximum likelihood (ML) was used to estimate the indirect effects.

## Results

**Figure 1:**
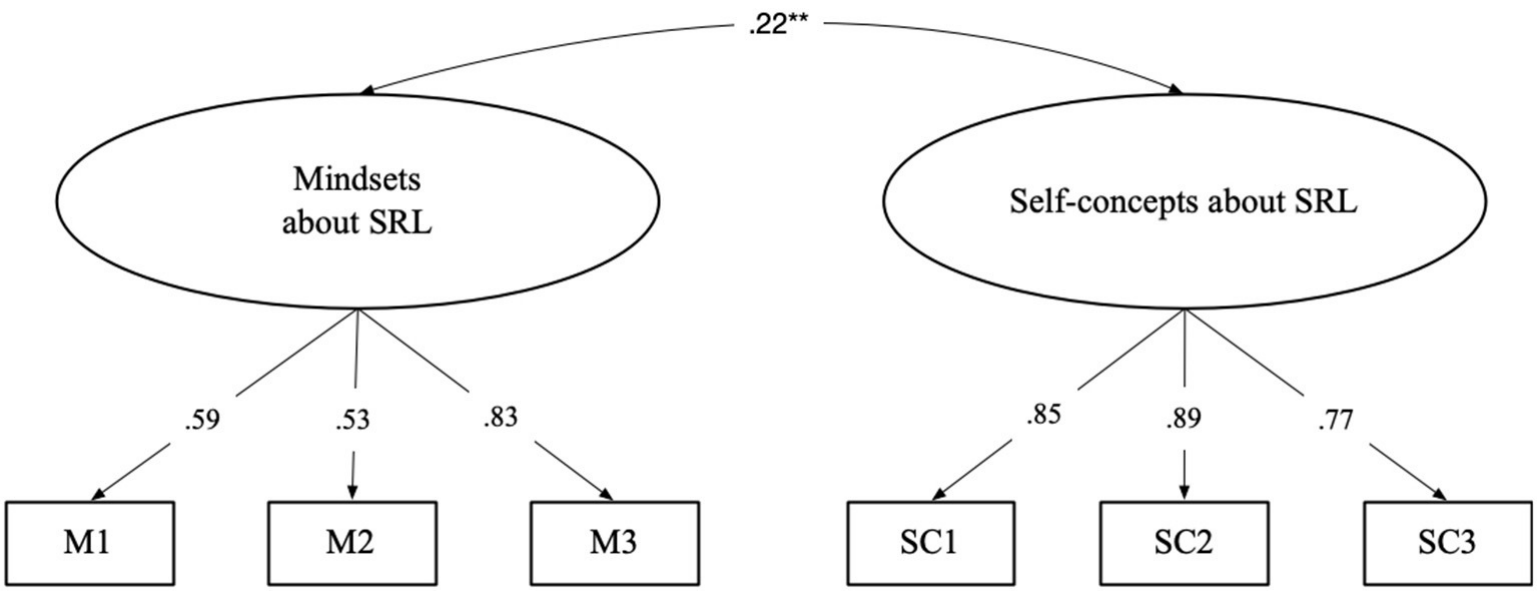
Confirmative factor analysis of mindsets and self-concepts about SRL.**p < 0.01.

First, to examine the relationship between mindset and self-concept about SRL, a two-dimensional model with two correlated latent factors was specified (see Figure 1). This two-dimensional CFA model indicated immediately acceptable fit values [$\chi_{\left(8\right)}^{2}$ = 15.049, *p* = 0.06, χ^2^/df = 1.881, CFI = 0.980, RMSEA = 0.060, and SRMR = 0.024]. As expected, the results indicated that the two self-theories about SRL are discriminatory and moderately related.

Second, the descriptive statistics and correlations between all the variables are outlined in Table 1. As expected, students' mindsets and self-concepts about SRL correlated positively with enjoyment, strategy knowledge, and academic achievement and correlated negatively with boredom. All the antecedents for academic achievement demonstrated significant positive relationships (mindset about SRL, self-concepts about SRL, strategy knowledge, and enjoyment) or negative relationship (boredom).

**Figure 2:**
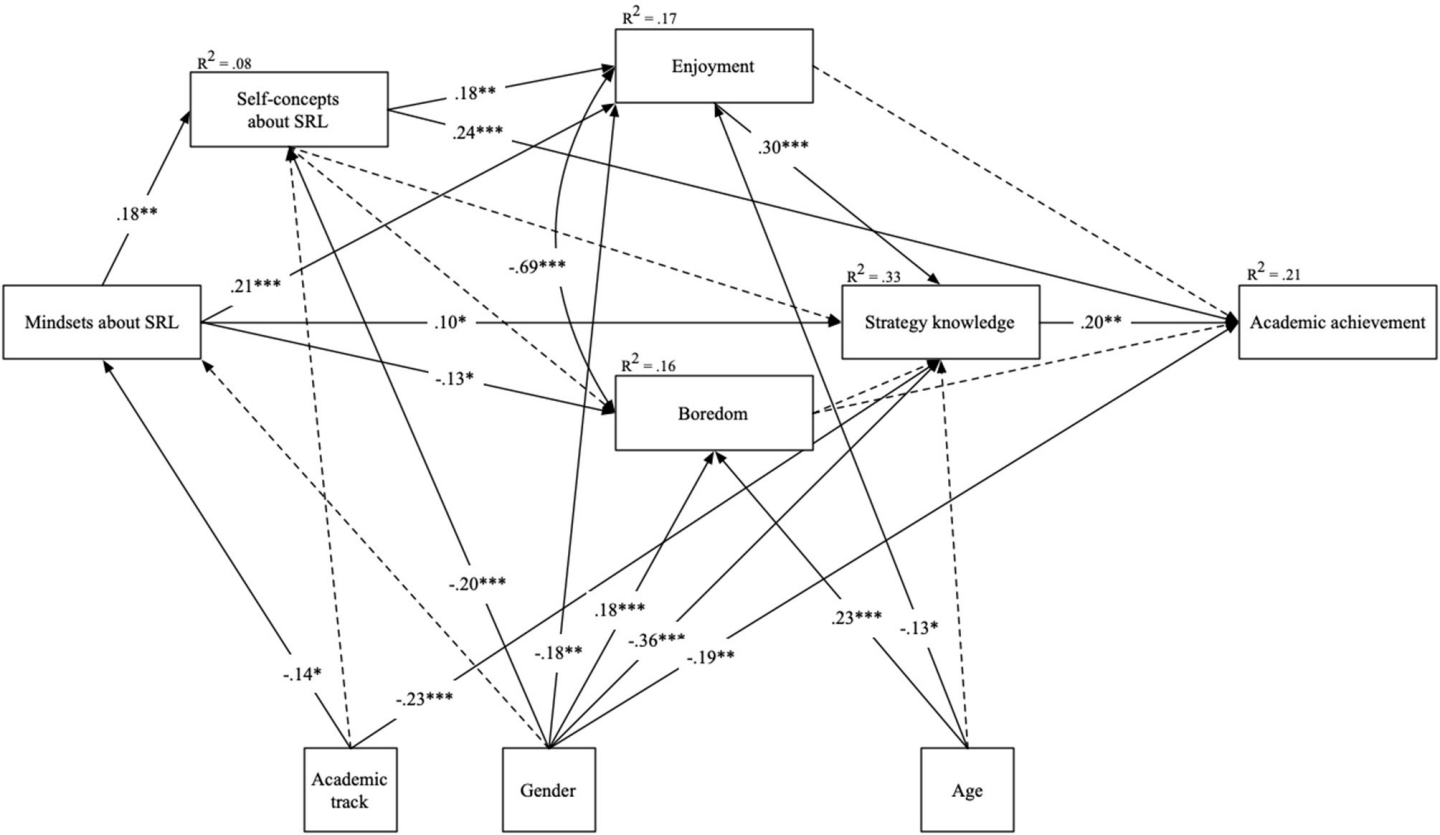
Path analysis model of associations between implicit theories of SRL, self-concept about SRL, emotions, strategy knowledge, and academic achievement. Continuous lines represent significant paths; dotted lines represent non-significant but estimated paths. Standardized regression coefficients are presented. Gender is coded as 1 = female; 2 = male. Academic track is coded as 1 = higher track; 2 = lower track. *p < 0.05. **p < 0.01. ***p < 0.001.

Based on theoretical assumptions and previous findings (Pekrun, 2006; Efklides, 2011), a path model was performed on the data to investigate the relationships between the variables in this study. All modeled paths are displayed in the model as no paths were removed (see Figure 2). The path model directly demonstrated an excellent fit to the data: $\chi_{\left(7\right)}^{2}$ = 2.459, *p* = 0.930, χ^2^/df = 0.351, CFI = 1.000, RMSEA = 0.000, and SRMR = 0.017. Mindsets about SRL positively related to self-concepts about SRL, enjoyment, and strategy knowledge. When students believed that SRL competencies are malleable they enjoyed learning at school more and had increased knowledge about strategies. In turn, mindsets about SRL negatively correlated with boredom, meaning that the students who adhered to a growth mindset about SRL found learning less boring than students who did not adhere to a growth mindset. The higher the students' self-concepts were about SRL, the more they reported that they enjoyed learning. In contrast, there was no significant correlation between self-concepts about SRL and boredom. Furthermore, students' self-concepts about SRL positively related to academic achievement. As expected, students' strategy knowledge also positively related to their academic achievement. Finally, whereas enjoyment positively related to strategy knowledge, boredom did not significantly correlate with strategy knowledge.

In terms of the covariates, female students reported higher self-concepts about SRL, gained higher strategy knowledge, reported higher enjoyment and lower boredom, and had higher levels of academic achievement than male students did. As expected, no gender differences were found for mindsets about SRL. Students from the lower academic track reported that they adhered more to a fixed mindset about SRL and demonstrated lower strategy knowledge than their peers from the higher academic track did. Finally, the older the students were, the less they reported enjoying learning at school and the more they reported higher levels of boredom. Altogether, all the variables within the model explained *R*^2^ = 0.21 (*p* < 0.001) of the variance in academic achievement.

We tested the mediation effects by examining the indirect effects of mindsets and self-concepts about SRL on enjoyment, strategy knowledge, and academic achievement. We conducted 1,000 bootstraps. The total direct, total indirect, and specific indirect effects are outlined in Table 2. The results revealed four significant indirect effects. When students more strongly endorse a growth mindset about SRL, they demonstrated higher strategy knowledge due to their higher self-concepts about SRL. Furthermore, when students more strongly endorsed a growth mindset about SRL, they experienced higher levels of academic achievement due to their higher self-concepts about SRL. Additionally, the higher the students' self-concepts were, the higher their strategy knowledge was due to their enjoyment of learning. Finally, the higher the students' perceived enjoyment of learning was, the higher their levels of academic achievement were due to their higher levels of strategy knowledge.

**Table 2:** Total effect, total indirect effects and specific indirect effect of mindsets about SRL, self-concept about SRL, enjoyment, strategy knowledge, and academic achievement.

**Hypothesized effects**	**Observed effects**	
	**Estimates**	**SE**
Mindsets > enjoyment		
Total effect M > EJ	**0.240**	**0.064**
Total indirect effect M > EJ	0.031	0.017
Specific indirect		
M > SC > EJ	0.031	0.017
Mindsets > strategy knowledge		
Total effect M > SK	**0.140**	**0.051**
Total indirect effect IT > SK	**0.054**	**0.023**
Specific indirect		
M > EJ > SK	**0.061**	**0.024**
Self-concepts > strategy knowledge		
Total effect SC > SK	0.090	0.055
Total indirect effect SC > SK	0.031	0.020
Specific indirect		
SC > EJ > SK	**0.052**	**0.025**
Mindsets > academic achievement		
Total effect M > AA	**0.098**	**0.029**
Total indirect effect M > AA	**0.098**	**0.029**
Specific indirect		
M > SC > AA	**0.039**	**0.019**
M > SK > AA	0.016	0.012
M > SC > SK > AA	0.002	0.001
M > EJ > SK > AA	0.002	0.003
M > SC > EJ > SK > AA	0.001	0.001
Self-concepts > academic achievement		
Total effect SC > AA	**0.252**	**0.059**
Total indirect effect SC > AA	0.029	0.018
Specific indirect		
SC > SK > AA	0.011	0.017
SC > EJ > SK > AA	0.010	0.006
Enjoyment > academic achievement		
Total effect EJ > AA	0.105	0.084
Total indirect effect EJ > AA	**0.054**	**0.027**
Specific indirect		
EJ > SK > AA	**0.054**	**0.027**

*Standardized effects are shown; statistical significance was determined using 1,000 bootstraps. Bold print for significant total effect, total indirect effects, and indirect effects. M, mindsets about SRL; SC, self-concepts about SRL; EJ, enjoyment; SK, strategy knowledge; AA, academic achievement*.

## Discussion

This study focused on two crucial self-theories about abilities that represent independent but related components of individuals' belief systems about SRL, which affect how individuals approach academic situations, how they perceive their knowledge and their abilities and respond to challenges within such situations (Dweck and Leggett, 1988). Mindsets and self-concepts create a system of meaning that sets goal-directed top-down and bottom-up SRL processes in motion and, thus, is the source of different SRL trajectories, emotions, motivation, and differences in academic achievement (Efklides, 2011; Burnette et al., 2013). In this study, we have assessed students' mindset and self-concept about SRL. We explored the relationship between students' mindsets and self-concepts about SRL and enjoyment, boredom, strategy knowledge, and academic achievement while controlling for students' genders, ages, and academic track. Overall, the results revealed that students' mindsets and self-concepts about SRL positively relate directly or indirectly to their enjoyment, strategy knowledge, and academic achievement. In the following sections, according to our research questions and hypotheses, we discuss this study's findings in more detail and draw conclusions for practice.

Our first research question addressed the relationship between mindsets and self-concepts about SRL. Our results revealed that the students' mindsets and self-concepts about SRL were separate but positively interrelated constructs, which confirmed our first hypothesis. Students with a growth mindset about SRL reported higher self-concepts about SRL than students with a fixed mindset about SRL. A possible explanation for this finding is that students with growth mindsets tend to perceive learning situations as opportunities to grow and expand their competencies (Dweck and Leggett, 1988). They consider failures and mistakes that they experience while learning and applying strategies to be feedback for their SRL, which can be developed through further practice. In contrast, students with a fixed mindset about SRL see SRL failures as a threat to their perceived competence in SRL. Thus, mindsets about SRL might also take on a protective function for students' self-concepts about SRL. However, researchers need to conduct longitudinal studies to investigate this assumption.

Our second research question focused on the relationship between mindsets and self-concepts about SRL, emotions, and strategy knowledge. We expected that mindsets and self-concepts about SRL would positively relate to enjoyment (Hypothesis 2a), negatively relate to boredom (Hypothesis 2b), and positively relate to strategy knowledge (Hypothesis 2c), which we were mostly able to confirm. The students' mindset about SRL positively related to strategy knowledge. This relationship can be explained using findings from other studies that have demonstrated that students with a growth mindset about intelligence more frequently implement strategies, demonstrate higher engagement in strategic behavior, and possess higher metacognitive awareness than students with a fixed mindset (Burnette et al., 2013; Yan et al., 2014). These learning behaviors support students' development of strategy knowledge (Karlen and Compagnoni, 2017; Chen et al., 2020; Hertel and Karlen, 2020). Mindsets and self-concepts about SRL both positively related to enjoyment about learning, and mindsets about SRL also reduced boredom. These results align with Pekrun's (2006) control-value theory, which assumes that beliefs and self-concepts are important antecedents for emotions because they increase an individual's perception of being in control of the learning process (Pekrun and Stephens, 2010).

Our second research question focused also on the association between emotions and strategy knowledge. We expected enjoyment to positively correlate to strategy knowledge (Hypothesis 2d) and boredom to negatively correlate to strategy knowledge (Hypothesis 2e). Our results only confirmed the positive relationship between enjoyment and strategy knowledge. A possible explanation for this finding might be that positive emotions such as enjoyment influence SRL more strongly than negative emotions such as boredom do (Pekrun et al., 2002). Students might also build up strategy knowledge with less use of strategy, but it is more effective when students show deliberate strategy practice because students enjoy learning. Additionally, the importance of students' enjoyment of learning is highlighted by the results that we found regarding how mindsets and self-concepts about SRL indirectly impact strategy knowledge via enjoyment. Moreover, we found that enjoyment mediated the effects of students' self-concepts about SRL on strategy knowledge. Our results align with several researchers' findings that stresses the importance of emotions as a relevant component of students' SRL (Pekrun et al., 2002). These results point out that in the promotion of SRL, it is also essential to support adaptive emotions and the regulation of negative emotions (Ben-Eliyahu and Linnenbrink-Garcia, 2015). Self-theories about abilities that support control and value of learning might play a crucial role here (Pekrun, 2006).

However, we do not know whether successful or less successful SRL might have influenced students' emotions about learning. Emotions affect students' SRL and achievement, but related experiences of success and failure can in turn influence students' emotions (Pekrun et al., 2017). Determining this fact would require a longitudinal analysis of the interplay between these processes. This might be an interesting question for future studies.

Our third question investigated whether students' mindsets and their self-concepts about SRL indirectly or directly relate to academic achievement. The results revealed that students with a growth mindset and higher self-concepts about SRL reached higher academic achievement than their peers who adhered to a fixed mindset and possessed lower self-concepts, which aligned with Hypothesis 4. Self-concepts about SRL related directly to academic achievement. This finding supports existing results that have demonstrated the significance of self-concepts for academic achievement in school (Lauermann et al., 2019; Möller et al., 2020). The effect of mindsets about SRL on academic achievement was indirect, so this study also contributes to a growing body of evidence that has demonstrated that mindsets may have mainly indirect or small direct impacts on students' academic achievements (Costa and Faria, 2018; Sisk et al., 2018). In line with this, other researchers have demonstrated that a growth mindset positively affects beneficial learning factors such as motivation, the perseverance of effort, and SRL, all of which are essential antecedents of academic achievement (Burnette et al., 2013; Priess-Groben and Hyde, 2017; Karlen et al., 2019). However, we found that mindset about SRL did not significantly affect academic achievement via strategy knowledge. Integrating further SRL variables could therefore be important for future studies on this topic. It may be important to examine the relationship between mindset, SRL, and performance by including further SRL variables.

By focusing on the results that concern the covariates in our study, we can discuss some interesting results. Our results revealed that the girls reported higher self-concepts about SRL and obtained greater strategy knowledge than the boys did. These results also confirm findings from previous studies that have repeatedly demonstrated that girls have more strategy knowledge than boys do for different age groups (Händel et al., 2013; Maag Merki et al., 2013). Researchers have demonstrated that self-concepts give rise to SRL behaviors during learning and thus support the acquirement of strategy knowledge (Roebers et al., 2012; Bakadorova et al., 2020), which creates favorable learning conditions for girls, at least concerning SRL. However, we need further studies that investigate a possible reciprocal relationship between successful SRL and the development of students' self-concepts about SRL.

Gender-specific differences were also found for emotions. Our results are consistent with existing evidence that demonstrates that boys report less enjoyment and more boredom about learning than girls do (King and dela Rosa, 2019). These emotional differences might exist because the boys reported lower self-concept about SRL, which leads to gender-linked appraisals that are related to learning (Pekrun, 2006). In other words, the difference in perceived competence provides a lower expectation of control and success, which in turn might negatively influence male students' emotions about learning. Further, our results revealed that boys obtained lower grades at school than girls. They not only perceived lower competence in (self-regulated) learning, they were also less successful than girls, which both might explain emotional differences. In line with this assumption, Pekrun et al. (2017) showed reciprocal effects between emotions and academic achievement. However, due to our cross-sectional design, we cannot make any statements on reciprocal effects.

This study also revealed that the students in the lower academic track were at risk in two ways. On the one hand, when compared with students in the higher academic track, they more frequently reported having a fixed mindset about SRL, which confirmed previous results concerning how at-risk students have a relatively fixed view of mindsets of intelligence (Warren et al., 2019). Simultaneously, we also found that academic track as a factor negatively correlated with strategy knowledge, which means that the students from the lower academic track possessed less strategy knowledge than their peers in the more academically demanding track did. This finding could be one possible explanation for why students in lower academic tracks may have more difficulties in school since both mindsets and strategy knowledge relate to academic achievement (Händel et al., 2013).

### Practical Implications

This study's findings have implications for interventions that are designed to support students' SRL in classrooms. When designing and conducting SRL training sessions in classes, researchers and educators should consider and encourage growth mindsets about SRL (Hertel and Karlen, 2020). Fixed theorists may have strategies in their repertoire but may not use them. This may occur because they think that smart people do not need strategies or because they become defensive when learning becomes challenging. In this context, teachers' feedback might play an important role by attributing effort to strategy use rather than ability (Rattan et al., 2012). In line with conceptual change research in the context of SRL an important step toward producing some change in learners' implicit belief is to make them explicit and make them the subject of discussion and reflection in learning (Lawson et al., 2019; Vosniadou et al., 2020). Our results also demonstrate that students in the lower academic track adhere to a fixed mindset about SRL and possess lower strategy knowledge than their peers in the higher academic track do. Interventions that focus on mindsets have proven to be particularly relevant for low-performing and disadvantaged students and represent an important contribution to increasing equality and educational opportunities for those students (Binning et al., 2019). Therefore, low-performing students could especially benefit from combined training that focuses on mindsets about SRL and SRL. Finally, our results demonstrate that boys can represent an at-risk group concerning the promotion of self-concepts about SRL and strategy knowledge in comparison to girls. To strengthen their self-concepts about SRL (male), students need to experience success in SRL. Helping students maximize control and value in SRL may benefit their learning and academic achievements. For example, teachers could explicitly provide strategies to help students improve their control and overcome challenges and support students' SRL or to provide adaptive support to students in SRL. Simultaneously, that notion that everyone can overcome challenges with effort and strategies could support higher control and value and, thus, support emotions that encourage SRL (Pekrun, 2006). Overall, it could be particularly beneficial if SRL training not only fosters strategies but also supports beliefs that might be consistent with SRL theory. A growth mindset classroom could support the notion that everyone can progress with effort, that the deliberate use of strategies and academic failures are an important part of everyone's learning process.

### Limitations

This study's first limitation was that nearly all the variables were measured simultaneously and that all the results are correlational. Grades (academic achievement) were obtained afterward from students' semester report cards. However, these grades represented students' school performances from their last semester. Our path analysis model was estimated based on previous empirical and theoretical assumptions about the relationships between all the involved variables (Pekrun, 2006; Efklides, 2011). The relationships between the variables could also be modeled differently. For example, when measuring proximal influencing factors for self-concepts, one should consider the reciprocal relationship between self-concepts and academic achievement (Möller et al., 2020). One must also recognize that this study's results came from a non-experimental field study with a rather small sample, which might explain the observed relatively low effects. These restrictions limit the generalizability of our results; another verification by a larger sample is needed. Finally, we focused on two key emotions (enjoyment and boredom) that have been identified as being particularly relevant to students' learning (Pekrun et al., 2017). However, other essential emotions relate to students' learning (e.g., anger, hope, etc.). Therefore, future studies might focus on a broader range of emotions and expand our knowledge about the relationship between self-beliefs, emotions, and SRL.

## Conclusion

This study confirms that students' mindsets and self-concepts about SRL create a belief system that is important to students' enjoyment and boredom, strategy knowledge, and academic achievement. Overall, our results revealed that investigating students' mindsets and self-concepts about SRL and their relationships with other SRL variables might be worthwhile. Mindsets and self-concepts about SRL have the potential to contribute to a better understanding of why students might be inclined to engage in goal-directed SRL processes such as the activation of strategy knowledge or the regulation of emotions. It might also be essential to identify beliefs that are not consistent with SRL (Vosniadou et al., 2020), which might stand in the way of applying strategies, enjoying learning, and developing strategy knowledge. Students who think SRL is a malleable ability and belief that they have enough competencies in SRL to overcome challenges might be more likely to seek out opportunities to apply strategies.

## Data Availability Statement

The raw data supporting the conclusions of this article will be made available by the authors, without undue reservation.

## Ethics Statement

The studies involving human participants were reviewed and approved by University of Applied Sciences and Arts Northwestern Switzerland. Written informed consent to participate in this study was provided by the participants' legal guardian/next of kin.

## Author Contributions

YK led in writing the manuscript, conceived the study, and collected and analyzed the data. CH conceived the study, collected and prepared the data, provided critical feedback, and revised the manuscript. AL evaluated the strategy knowledge test, coded the students' answers, and proofread the manuscript. FS evaluated the strategy knowledge test, coded the students' answers, provided critical feedback, and revised the manuscript. All authors contributed to the manuscript and approved the submitted version.

## Conflict of Interest

The authors declare that the research was conducted in the absence of any commercial or financial relationships that could be construed as a potential conflict of interest.
